# India Hypertension Control Initiative: decentralization of hypertension care to health wellness centres in Punjab and Maharashtra, India, 2018–2022

**DOI:** 10.1186/s12913-024-11354-9

**Published:** 2024-08-02

**Authors:** Tejpalsinh A. Chavan, Mogan Kaviprawin, Manikandanesan Sakthivel, Navneet Kishore, Padmaja Jogewar, Sandeep Singh Gill, Abhishek Kunwar, Kiran Durgad, Amol B. Wankhede, Vishwajit Bharadwaj, Suhas N. Khedkar, Lalit Sarode, Bidisha Das, Sampada D. Bangar, Vettrichelvan Venkatasamy, Ashu Gupta, Mosoniro Kriina, Ashish Krishna, Anupam Khungar Pathni, Swagata K. Sahoo, Ganeshkumar Parasuraman, Roopa Shivashankar, Pragati Pragya, Meenakshi Sharma, Prabhdeep Kaur

**Affiliations:** 1 Division of Noncommunicable Diseases (NCD), Indian Council of Medical Research (ICMR) - National Institute of Epidemiology (NIE), Chennai, India; 2 India Hypertension Control Initiative (IHCI) Project, District NCD Cell, World Health Organization (WHO), Pune, India; 3 IHCI Project, State NCD Cell, WHO, Chandigarh, Punjab India; 4 State NCD Cell, Directorate of Health Services, Government of Maharashtra, Mumbai, India; 5 State NCD Cell, Department of Health and Family Welfare, Government of Punjab, Chandigarh, India; 6 Department of Noncommunicable Diseases, WHO Country Office, New Delhi, India; 7 IHCI Project, State NCD Cell, WHO, Mumbai, Maharashtra India; 8 IHCI Project, District NCD Cell, WHO, Bhandara, Maharashtra India; 9 IHCI Project, District NCD Cell, WHO, Satara, Maharashtra India; 10 IHCI Project, District NCD Cell, WHO, Wardha, Maharashtra India; 11 IHCI Project, District NCD Cell, WHO, Bhatinda, Punjab India; 12 ICMR-National AIDS Research Institute, Pune, India; 13 Resolve to Save Lives, New Delhi, India; 14 ICMR, New Delhi, India

**Keywords:** India, Hypertension, Non-communicable diseases, Patient-centric care, Decentralization, Blood pressure, Primary health care, Implementation, Health system

## Abstract

**Introduction:**

The India Hypertension Control Initiative (IHCI) emphasizes decentralized patient-centric care to boost hypertension control in public healthcare facilities. We documented the decentralization process, enrolment pattern by facility type, and treatment outcomes in nine districts of Punjab and Maharashtra states, India, from 2018–2022.

**Methods:**

We detailed the shift in hypertension care from higher facilities to Health and Wellness Centres (HWCs) using the World Health Organization (WHO) health system pillar framework. We reviewed hypertension treatment records in 4,045 public facilities from nine districts in the two states, focusing on indicators including registration numbers, the proportion of controlled, uncontrolled blood pressure (BP), and missed visits among those under care.

**Results:**

The decentralization process involved training, treatment protocol provision, supervision, and monitoring. Among 394,038 individuals registered with hypertension from 2018–2021, 69% were under care in 2022. Nearly half of those under care (129,720/273,355) received treatment from HWCs in 2022. Care of hypertensive individuals from district hospitals (14%), community health centres (20%), and primary health centres (24%) were decentralized to HWCs. Overall BP control rose from 20% (4,004/20,347) in 2019 to 58% (157,595/273,355) in 2022, while missed visits decreased from 61% (12,394/20,347) in 2019 to 26% (70,894/273,355) in 2022. This trend was consistent in both states. HWCs exhibited the highest BP control and the lowest missed visits throughout the study period compared to other facility types.

**Conclusion:**

We documented an increase in decentralized access to hypertension treatment and improved treatment outcomes over four years. We recommend operationalizing hypertension care at HWCs to other districts in India to improve BP control.

**Supplementary Information:**

The online version contains supplementary material available at 10.1186/s12913-024-11354-9.

## Introduction

Uncontrolled hypertension, a significant risk for cardiovascular disease (CVD) deaths in India, accounts for one-third of total fatalities [1, 2]. In 2015, only one-tenth of the 200 million individuals with hypertension had controlled blood pressure (BP). The India Hypertension Control Initiative (IHCI), launched in 2017, adopted the World Health Organization (WHO) HEARTS package, aiming for a 25% relative decrease in hypertension prevalence by 2025 and aligning with the Global Monitoring Framework [3–5].

The IHCI employs a decentralized strategy inspired by effective HIV and tuberculosis programs, shifting clinical responsibilities from specialists to community-level care providers for enhanced accessibility [6–9]. In India's healthcare system, District Hospitals (DH) function as referral centres within districts; hence, they serve as the most centralized facility. The next level includes Community Health Centres (CHCs), followed by Primary Health Centres (PHCs). Health and Wellness Centres (HWCs) are the most decentralized health facilities, closer to the communities. In the IHCI, an individual with hypertension initially registered in a DH, CHC, PHC, or HWC is transferred to a CHC, PHC, or HWC near their residence for subsequent follow-up [10].

Initially launched in five states, the IHCI focused on five strategies: standard treatment protocol, uninterrupted drug supply, task sharing, patient-centric care, and an information system to track key indicators [10]. In 2018, HWCs were established by the government under the flagship program "Ayushman Bharat." Subsequently, in 2018, the IHCI became operational in nine districts across Punjab and Maharashtra. In 2019, a digital system for recording registration, treatment outcomes, and follow-up was introduced in these two states, whereas other IHCI states continued with paper-based systems.

The extent of decentralized care, outcomes, and digital system utilization remains unclear. The documented evidence of the success and challenges of decentralization will allow policymakers to make informed decisions regarding expanding or optimizing hypertension programs.

We documented the decentralization process for hypertension care to HWCs. We assessed enrolment and treatment outcomes changes across facilities from 2018 to 2022 and examined the associations between the decentralization process and BP control.

## Methods

### Study design, population and setting

We described and documented the operationalization of hypertension-related services and decentralization process at HWCs in the two states using the WHO health system's six pillars framework: 1. Governance; 2. Financing; 3. Human resources; 4. Health information systems; 5. Availability of drugs, BP apparatus, and treatment protocols, and 6. Service delivery, including stakeholder engagement, training, registration, and supportive supervision [11].

We conducted a retrospective cohort study using secondary data from the IHCI for five districts in Punjab (Bathinda, Gurdaspur, Hoshiarpur, Mansa, and Pathankot) and four in Maharashtra (Bhandara, Satara, Sindhudurg, and Wardha) during 2018–2021.

### Operational definitions

#### Hypertension diagnosis

Systolic BP (SBP) ≥ 140 mmHg or Diastolic BP (DBP) ≥ 90 mmHg on two different days. However, if the SBP is ≥ 160 mmHg or DBP is ≥ 100 mmHg at the first reading, a second reading should be taken on the same day to establish the diagnosis [12].

#### Hypertension registration

Individuals diagnosed with hypertension by a medical officer, including those newly screened and diagnosed or already taking antihypertensive medications.

#### Individuals with hypertension under care

Individuals with hypertension who had at least one visit to a health care facility between 1st April and 31st March every year during the study period, excluding deaths and lost to follow-up.

#### BP under control

SBP < 140 mmHg and DBP < 90 mmHg during the most recent visit, between 1st January and 31^st^ March of every year during the study period, among individuals under care.

#### BP not under control

SBP ≥ 140 mmHg or DBP ≥ 90 mmHg during the most recent visit between 1^st^ January and 31^st^ March every year during the study period among individuals under care.

#### Missed visit

Individuals with hypertension under care who had not recorded a visit in one reporting quarter between 1st January to 31st March every year during the study period among individuals under care [10].

#### Lost to follow-up

Individuals with hypertension who did not have any follow-up visits over 12 months between 1st April and 31st March every year during the study period [10].

#### Retention in care

Individuals with hypertension who visited a health care facility between 1st January and 31st March every year during the study period, irrespective of their BP status.

### Data collection and management

We reviewed IHCI guidelines and government documents and conducted field observations of project teams. We abstracted the deidentified line list data of individuals with hypertension registered under IHCI from 4,054 health facilities in the nine districts from the "simple app" database [13]. "Simple app" is an Android app used for longitudinal reporting of hypertension treatment outcomes. The progress of individuals under care can be tracked in real-time, with performance monitored daily or monthly. Reports are automatically generated, saving time previously spent compiling and verifying paper records. Simple was recommended for use in busy health facilities based on its usability and user-friendliness [13, 14]. We cleaned the data in Microsoft Excel. Variables of interest included the number of registrations, annual treatment outcomes, last visit date, baseline, and BP measurement on the most recent visit.

### Data analysis

We analyzed the trend in enrolment of hypertension individuals by year, state, district, and facility type. Using a Sankey chart [15], we visualized the flow or allocation of individuals with hypertension from the enrolment facility to the facility that individuals visited during their most recent follow-up. Although individuals with hypertension undergo follow-up visits throughout all quarters of the year, the project guidelines include an assessment of treatment outcomes based on the most recent visit during the first quarter of each year to enable comparisons across states that use different information systems, including paper-based systems.

Treatment outcomes included controlled BP, uncontrolled BP, and missed visits. We analyzed the data by age, gender, comorbidities, and BP levels. The baseline and recent BP measurements were categorized as control, grade one (SBP as 140–159 mmHg or DBP as 90–99 mmHg), and grade two (SBP ≥ 160 mmHg or DBP ≥ 100 mmHg).

We examined the association between the facility type where the individuals received care and BP control using a Generalized Estimating Equation (GEE) with an unstructured correlation structure using a four-timepoint (2019–2022) longitudinal model [16]. GEE was employed due to the clustering of outcomes at the individual level (BP status). The outcome was the BP status during each year from 2019 to 2022. We adjusted for year of assessment, age, gender, baseline BP control status, diabetes, prior heart attack, prior stroke, prior chronic kidney disease (CKD), treatment for hypertension during registration, and the state of residence. We measured the association using relative risk (RR) with a 95% confidence interval (CI). We considered *P values* < *0.05* as statistically significant. We used Stata version 17 to analyze the data [17].

## Results

### Process of decentralization

- Governance and financing: The Government of India outlined guidelines for HWC establishment, acting as an implementation blueprint. Using the guidelines, the state governments started establishing the HWCs in a phased manner. State and district health coordinators oversee HWC rollout. A feedback mechanism monitors indicators at state and national levels. Regular stakeholder meetings tackle health system barriers. A shared budget between federal and state governments supports HWC. The state Non-Communicable Disease (NCD) cell allocates funds for training, diagnostics, and drugs. The stakeholders were involved in operationalizing hypertension services at the state and district levels by developing implementation plans. The close coordination between the HWC implementation team and the state NCD cell, with support from the IHCI team, enabled the development of guidelines and implementation plans.
- Human resources: The HWCs were supported by Community Health Officers (CHOs) with technical support from the IHCI team. The state health department enlisted nurses as CHOs, supported by Auxiliary Nurse Midwives (ANMs) and Accredited Social Health Activists (ASHAs). ASHAs, one for every 1000 community members, connect the community with the public health system, promote awareness and facility use, and contribute to decentralizing hypertension care. CHOs, ANMs, and healthcare staff were the regular staff under the National Programme for Prevention and Control of Non-Communicable Diseases (NP-NCD). They received hypertension training from the additional project staff, including the Cardiovascular Health Officers (CVHO) and Senior Treatment Supervisors (STSs). The training included treatment protocols, supervised drug refills, accurate BP measurement, drug stock management, teleconsultation, and data recording via the "simple app."
- Service delivery: Hypertension services were phased in HWCs across nine districts from 2018–2021, witnessing an increase from 37 in 2018 to 261 in 2019, further expanding to 1,374 in 2020 and reaching a total of 3,412 in 2021. In 2022, the HWCs offered 13 services, encompassing screening for NCDs and follow-up care, including drug refills for individuals undergoing treatment, including hypertension.
- Treatment protocols, availability of drugs, and blood pressure apparatus: IHCI adapted treatment protocols based on WHO's Technical Package for Cardiovascular Disease Management in Primary Health Care (HEARTS). The protocol encompassed amlodipine, telmisartan, and chlorthalidone as the designated drugs, but treatment methods varied across states [18]. Before IHCI, drugs could only be refilled at DHs, CHCs, or PHCs. The supply chain was strengthened by a collaborative effort between the states and IHCI to provide refills for antihypertensive drugs at HWCs. The project team developed curated interventions for improving supply chain efficiency and extended continuous technical support. The interventions included advocacy for procuring antihypertensive drugs, which were part of the adapted protocol, and easy-to-use tools for forecasting and stock maintenance [19]. Regular facility-level assessments were established to streamline drug procurement and distribution monthly. Validated models of professional BP monitors were used. An Arm-in type of Omron BP monitor was used in a few DHs, which caters to high patient loads.
- Health information systems: In the nine project districts, a digital system, the "simple app," was used to register individuals under care, follow up on treatment (drug refills) and modification, monitor retention, and track the outcomes over time.
  - *Registration/assigning diagnosed individuals with hypertension*: Staff at higher-level facilities (DHs, CHCs, and PHCs) could use the "simple app" to assign a facility nearer to the individual's home, schedule an appointment, and then issue a QR-coded card to continue treatment [13].
  - *Follow-up and issuing refills*: BP measurement and medications were facilitated during follow-ups through personal information or QR code scanning on treatment cards. The drug refills covered one month. CHOs sought teleconsultation with the PHC doctor or a panel of district/state-level doctors to modify or escalate medications.
  - *Retention in care*: The "simple app" provided a real-time list of registered individuals under care who were overdue for follow-up. Individuals under care at higher facilities were encouraged to continue treatment at their nearest HWC. Individuals under care at HWCs would receive a reminder SMS/phone call/visit from a CHO or an ASHA.
  - *Supportive supervision and reviews*: The "simple app" dashboard displayed real-time registrations and treatment outcomes. It helped identify facilities with poor outcomes for supportive supervision. Regular review meetings were held by district and state officials to monitor progress.

### Registrations

**Fig. 1:**
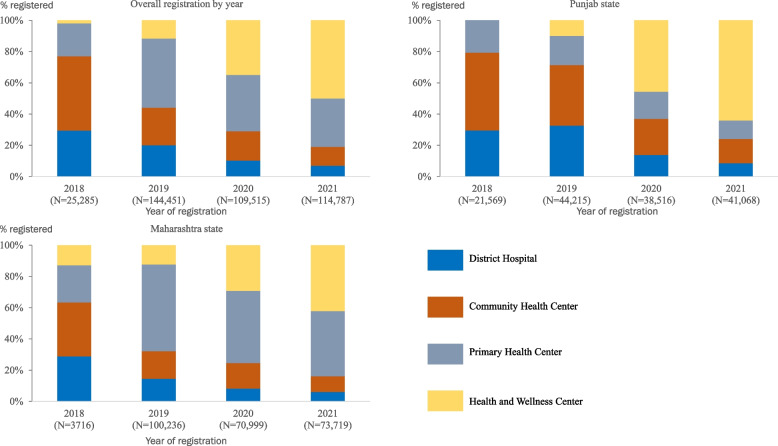
Registration of individuals with hypertension by facility type, year and state, Punjab and Maharashtra, India, 2018–21 (*N* = 394,038)

A total of 394,038 individuals with hypertension were registered under IHCI in nine districts of Punjab and Maharashtra states from 2018 to 2021. Among them, 145,368 (37%) were registered in Punjab and 248,670 (63%) in Maharashtra. The proportion of registrations in HWCs in Punjab increased from 10% (4,387/44,215) in 2019 to 64% (26,313/41,068) in 2021, while in Maharashtra, HWC registrations increased from 13% (477/3,716) in 2018 to 42% (30,984/73,719) in 2021. The registrations in PHCs in Maharashtra increased from 24% (885/3,716) in 2018 to 42% (30,779/73,719) in 2021. The overall registrations decreased in DHs (30% [7,460/25,285] in 2018 to 7% [8,060/114,787] in 2021) and CHC (48% [12,011/25,285] in 2018 to 12% [13,796/114,787] in 2021). The pattern was similar in both states (Fig. 1).

Of the 394,038 individuals registered with hypertension, 273,355 (69.3%) were under care in 2022, and 120,683 (30.7%) were lost to follow-up (LFTU). Among those under care, 72% were aged ≥ 55 years, and 60% were females; this pattern was similar in both states. Approximately one-third of individuals were already taking antihypertensive medications when registered (Additional file 1).

### Key indicators of decentralization

**Fig. 2:**
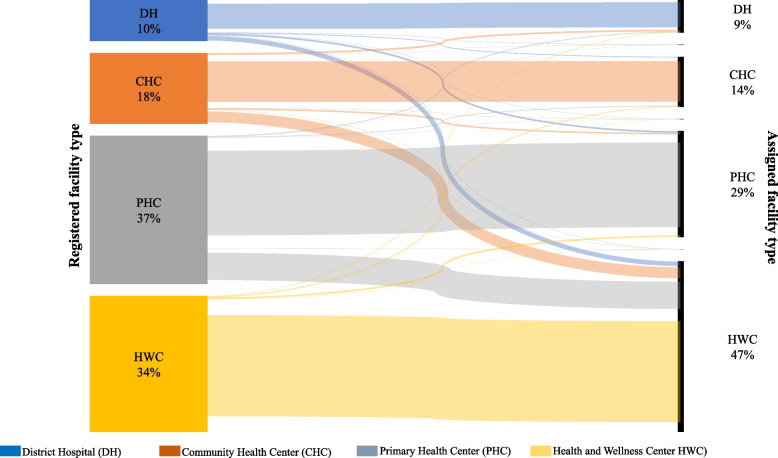
Proportion of individuals with hypertension at registration and recent visit by type of facility, Punjab and Maharashtra, India, 2018–22 (*N* = 273,355)

Of the 273,355 individuals with hypertension under care in 2022, two-thirds were in Maharashtra state. Among those under care in 2022, 47% (129,720/273,355) received or sought treatment at HWCs, followed by PHCs (29%, [80,543/273,355]) and CHCs (14%, [38,177/273,355]). Of the 179,535 individuals registered in DHs, CHCs, and PHCs, 38,189 (21%) shifted to HWCs. Overall, 14% (3,941/28,348) of individuals registered in DHs were assigned to HWCs. The pattern was similar in both states. Of the registrations in CHCs, 25% (5,456/21,717) in Punjab and 16% (4,244/27,119) in Maharashtra received treatment at HWCs during the most recent visit in 2022. Among individuals registered in PHCs, one-fifth (2,903/13,761) in Punjab and a quarter (21,645/88,590) in Maharashtra sought treatment at HWCs (Fig. 2, Additional file 2).

### Treatment outcomes

**Fig. 3:**
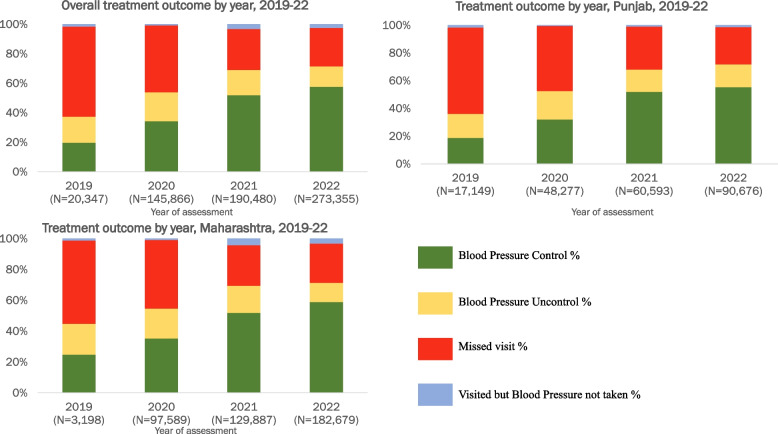
Treatment outcome among individuals with hypertension under care by year, Punjab and Maharashtra, India, 2019–22

In 2020, retention in care varied from 52% (14,527/28,124) in DH to 58% (11,954/20,591) in HWC. By 2022, overall retention improved to 72% (195,480/273,355). HWCs showed the highest retention among all facilities during the study period. Overall BP control increased from 20% (4,004/20,347) in 2019 to 58% (157,595/273,355) in 2022. The missed visits decreased from 61% (12,395/20,347) in 2019 to 26% (70,894/273,355) in 2022. The pattern was similar in both states (Fig. 3).

**Fig. 4:**
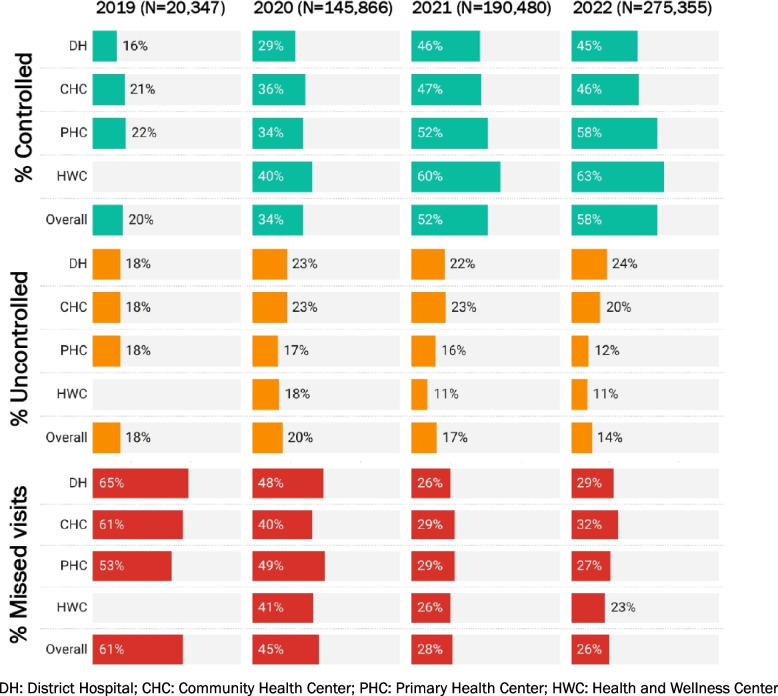
Treatment outcome among individuals with hypertension under care by facility type and year, Punjab and Maharashtra, India, 2019–22

BP control was highest at HWCs throughout the study period, with missed visits declining from 41% (8,545/20,591) in 2020 to 23% (29,994/129,720) in 2022. The missed visits remained lowest at HWCs throughout the study period (Fig. 4, Additional file 3).

Compared to individuals below 45 years, the risk of uncontrolled BP was higher among those aged 55–69 years (aRR = 1.02; 95% CI, 1.01–1.05) (Additional file 4). The risk of having uncontrolled BP was higher among those who received treatment at DHs [aRR = 1.50; 95% CI, 1.48–1.52], CHC [aRR = 1.38; 95% CI, 1.36–1.40] and PHCs [aRR = 1.25; 95% CI, 1.23–1.26] compared to those who received treatment at HWCs after adjusting for various covariates including age (Table 1).

**Table 1:** Association between facility type and hypertension control status, Punjab and Maharashtra, India, 2019–22

	Controlled n (%)	Uncontrolled n (%)	RR (95%CI)	*p*-value	ARR^a^	*p*-value
HWC	276,328 (79.2)	72,720 (20.8)	ref		ref	
PHC	110,720 (72.4)	42,264 (27.6)	1.34 (1.32–1.35)	< 0.01	1.25 (1.23–1.26)	< 0.01
CHC	53,537 (66.7)	26,759 (33.3)	1.59 (1.57–1.61)	< 0.01	1.38 (1.36–1.40)	< 0.01
DH	32,639 (64.7)	17,838 (35.3)	1.71 (1.69–1.74)	< 0.01	1.50 (1.48–1.52)	< 0.01

*DH* District Hospital, *CHC* Community Health Centre, *PHC* Primary Health Centre, *HWC* Health and Wellness Centre, *CI* Confidence interval, *ARR* Adjusted relative risk

^a^Adjusted for year of assessment, age (continuous), gender, baseline hypertension control status, diabetes, prior heart attack, prior stroke, prior CKD, taking hypertension drug at registration and state

## Discussion

We documented the process of decentralizing the individuals with hypertension to HWCs and how it affected the retention in care and BP control. Though HWCs were established by the state government, IHCI catalyzed the decentralization process. Our study showed a gradual increase in hypertension enrolment in HWCs over four years. One-fifth of individuals registered in higher-level facilities transitioned to HWCs for hypertension care in the IHCI-implemented states of Punjab and Maharashtra, India. The retention in care and BP control were higher in HWCs than in higher-level health facilities. Although the treatment protocols employed in the two states differed, the treatment outcomes exhibited a similar increase in both states [18]. This suggests that the decentralization approach may have effectively enhanced treatment outcomes independent of the specific treatment protocols used.

We observed the preference for HWCs among individuals with hypertension under care in our study setting. The preference could possibly be due to proximity, ease, lower cost of travel, less waiting time, and the ability to communicate with the health providers. This was consistent with the Government of India’s policy to invest resources in HWCs to improve access to primary care at the village level [20]. The public sector hospitals are often located in the district headquarters, limiting access to the people living in rural areas. A study from one of the project sites in Punjab, India, documented that nearly half of the patients who did not return to care were continuing treatment in the private sector, possibly due to proximity and rapport [21]. Our results were consistent with other studies in low- and middle-income countries (LMICs). In Bangladesh, 53.5% of the 891 individuals surveyed in the Household Income and Expenditure Survey (HIES) preferred healthcare facilities based on proximity and affordability [22]. A study in Rwanda compared the follow-up rates of higher and primary-level facilities. It showed that LFTU in referral hospitals was higher than in decentralized facilities. The patients traveled significantly longer distances than those treated at primary-level centres (10.4 km compared to 2.9 km, respectively) [8]. A patient-centric approach improved hypertension care across various settings. In developed countries like Canada, Iceland, and South Korea, the age-standardized hypertension treatment was over 70%, and control was observed at over 50% in 2019, which could be attributed to the quality of care, which includes high drug adherence, patient satisfaction, availability of drugs and logistics, and provider adherence to standard treatment guidelines, including prescribing practices [2, 23]. We need to improve hypertension control by locally adapting the best practices from other countries.

The availability of hypertension care at HWCs improved hypertension control in the study sites. The combination of treatment protocols, improved drug availability, and real-time HWC level monitoring enabled good quality hypertension care by a trained CHO. Our experience is consistent with primary care-based models in other LMICs. A comprehensive primary care model of care for hypertension in Cuba and Peru documented similar improvements in BP control [7, 24]. The provision of the standard treatment protocol to specially trained nurses at the primary level improved hypertension control [7]. A systematic review of nursing interventions targeting NCD outcomes demonstrated that patient outcomes improved due to nursing health education interventions [25]. The training empowered CHOs to perform BP measurements, refill antihypertensive drugs, and engage in teleconsultations with medical officers for treatment protocol adjustments without the need for referrals or unnecessary trips to higher facilities. The nurse-led hypertension care in Uganda clinic settings showed a mean reduction in SBP of 9.5 mmHg among 54 patients [26]. The components included health education on lifestyle modifications, treatment protocols, and follow-up of patients using text message reminders [26]. A study from South Africa highlighted the importance of patient education, where patients reported they were not adequately equipped for self-management [27]. Non-physician health workers can fill this gap and improve patient care. A Rwanda study observed that nurse-led hypertension care with treatment protocol availability improved control rates (40%, [20/50]) compared to higher facilities (32%, [20/62]) [8]. Considering the lack of adequate doctors in rural areas, nurse-based hypertension care at the peripheral level might help expand treatment coverage, even in remote regions of India. We acknowledge that other challenges in the health system influence patient care and treatment outcomes, even if HWC can provide decentralized care. We have earlier documented the challenges in the drug supply chain at various levels, such as estimation of requirements, procurement, and distribution [19]. A health system preparedness assessment of 39 PHCs in Karnataka, Southern India, revealed that 62% of the PHCs did not have a functional laboratory, 31% lacked a pharmacist for drug distribution, and 30% were without a medical officer. Additionally, over 60% of the PHCs reported stockouts of basic NCD drugs for more than one month [28].

The IHCI project period included the COVID-19 pandemic, significantly impacting health services. However, policy interventions during the COVID-19 pandemic facilitated decentralized care for NCDs. During the COVID-19 pandemic, based on the recommendations of the Ministry of Health & Family Welfare, Government of India, IHCI districts adopted a mitigation strategy of decentralized care and implemented the prescription of anti-hypertension drugs for extended periods (2–3 months) and community-level distribution of medications to patients with NCDs was done through HWCs. The intervention resulted in over 80% of hypertension patients receiving medications during the pandemic [29]. Once the pandemic was over, the patients were directed to collect refills at the HWCs for sustainability.

The real-time patient tracking through an app-based information system in a programmatic context was a strength of our study. Nurses documented BP readings and medications during patient clinic visits using the digital system, eliminating the need for paper-to-digital data transfer and reducing errors. This improved health information system verified BP recording timing and visit times precisely. However, our study is subject to several limitations. Firstly, one-third of individuals enrolled with hypertension were LTFU, and we could not assess the treatment outcomes among them. Secondly, the quality of care provided might have differed across various settings, contingent on factors such as the proficiency and training of healthcare staff and the accessibility of antihypertensive medications, which can affect the study's outcomes. Thirdly, our analysis focused on individual-level factors influencing treatment outcomes rather than facility-level factors such as drug availability. Nevertheless, we closely monitored and tracked the facility-level interventions of the IHCI, particularly emphasizing actions to ensure the availability of sufficient drugs [10]. We did not document the effect of decentralization on out-of-pocket expenditure, which needs to be explored in further studies. Hence, additional research could explore the financial implications of the initiative on individuals and households. Although variables such as socioeconomic status, lifestyle factors (diet, exercise), and medication adherence could be potential confounders for treatment outcomes, the information was not collected routinely and documented in the information system. Additionally, future research could explore the broader social determinants of health and patient perspectives on self-management in hypertension care. Furthermore, there is a need to consider the unique healthcare needs of diverse population groups, including tribal communities, in decentralizing hypertension care.

In conclusion, access to hypertension care, especially BP measurements and refills, led by non-physician health workers, improved retention in treatment and BP control. However, we need further research to understand the feasibility of titrating medications at peripheral health facilities. We recommend scaling decentralized care to other districts of India to expand access to hypertension treatment.

### Supplementary Information

Supplementary Material 1.

Supplementary Material 2.

Supplementary Material 3.

Supplementary Material 4.

## Data Availability

The datasets used and/or analyzed during the current study are available from the corresponding author upon reasonable request.
