# Supplementary material for: India Hypertension Control Initiative: decentralization of hypertension care to health wellness centres in Punjab and Maharashtra, India, 2018–2022
Source: BMC Health Serv Res. 2024 Aug 2;24:884. doi: 10.1186/s12913-024-11354-9 (PMC11297667; doi:10.1186/s12913-024-11354-9)
Supplement: Supplementary file 1 — Supplementary Material 1. [file 12913_2024_11354_MOESM1_ESM.docx]

| **Additional file 1: Distribution of individuals with hypertension under care by person characteristics, Punjab and Maharashtra, India, 2022** | | | | | | | | | | |
| --- | --- | --- | --- | --- | --- | --- | --- | --- | --- | --- |
| **Parameters** |  | **Punjab** | | | **Maharashtra** | | | **Total** | | |
|  |  | **N** | **n** | **%** | **N** | **n** | **%** | **N** | **n** | **%** |
| **Age group** | <45 | 90,676 | 8882 | 10 | 1,82,679 | 12,094 | 7 | 2,73,355 | 20976 | 8 |
|  | 45-54 | 90,676 | 20755 | 23 | 1,82,679 | 34,880 | 19 | 2,73,355 | 55635 | 20 |
|  | 55-69 | 90,676 | 42249 | 47 | 1,82,679 | 90,848 | 50 | 2,73,355 | 133097 | 49 |
|  | >=70 | 90,676 | 18790 | 21 | 1,82,679 | 44,857 | 25 | 2,73,355 | 63647 | 23 |
| **Gender** | Female | 90,676 | 58954 | 65 | 1,82,656 | 1,05,703 | 58 | 2,73,332 | 164657 | 60 |
|  | Male | 90,676 | 31707 | 35 | 1,82,656 | 76,953 | 42 | 2,73,332 | 108660 | 40 |
|  | Transgender | 90,676 | 15 | 0 | 1,82,656 | 23 | 0 | 2,73,332 | 38 | 0 |
| **Diagnosed with diabetes** | | 90,674 | 19048 | 21 | 1,82,670 | 40,893 | 22 | 2,73,344 | 59941 | 22 |
| **Prior heart attack** | | 90,674 | 1351 | 1 | 1,82,670 | 1,946 | 1 | 2,73,344 | 3297 | 1 |
| **Prior stroke** | | 90,674 | 594 | 1 | 1,82,670 | 1,823 | 1 | 2,73,344 | 2417 | 1 |
| **Chronic kidney disease** | | 90,674 | 348 | 0 | 1,82,670 | 410 | 0 | 2,73,344 | 758 | 0 |
| **Already on hypertension**  **medicine during registration** | | 90,674 | 23524 | 26 | 1,82,670 | 61,694 | 34 | 2,73,344 | 85218 | 31 |
| **Baseline Blood**  **Pressure category** | Controlled | 48,413 | 13,156 | 27 | 1,13,013 | 64,924 | 57 | 1,61,426 | 78080 | 48 |
|  | Grade-1* | 48,413 | 20,874 | 43 | 1,13,013 | 29,581 | 26 | 1,61,426 | 50455 | 31 |
|  | Grade-2** | 48,413 | 14,383 | 30 | 1,13,013 | 18,508 | 16 | 1,61,426 | 32891 | 20 |
| **Followup Blood**  **Pressure category** | Controlled | 72,397 | 64,524 | 89 | 1,54,903 | 1,45,776 | 94 | 2,27,300 | 210300 | 93 |
|  | Grade-1 | 72,397 | 5,827 | 8 | 1,54,903 | 6,956 | 4 | 2,27,300 | 12783 | 6 |
|  | Grade-2 | 72,397 | 2,046 | 3 | 1,54,903 | 2,171 | 1 | 2,27,300 | 4217 | 2 |

*Systolic blood pressure between ≥140mmHg and ≤159mmHg, Diastolic blood pressure between ≥90mmHg and ≤99mmHg; **Systolic blood pressure between ≥160mmHg, Diastolic blood pressure between ≥100mmHg
