# Supplementary material for: India Hypertension Control Initiative: decentralization of hypertension care to health wellness centres in Punjab and Maharashtra, India, 2018–2022
Source: BMC Health Serv Res. 2024 Aug 2;24:884. doi: 10.1186/s12913-024-11354-9 (PMC11297667; doi:10.1186/s12913-024-11354-9)
Supplement: Supplementary file 2 — Supplementary Material 2. [file 12913_2024_11354_MOESM2_ESM.docx]

| **Additional file 2: Proportion of individuals with hypertension at registration and recent visit by type of facility among under care, Punjab and Maharashtra, India, 2018-22 (N=273,355)** | | | | | | |
| --- | --- | --- | --- | --- | --- | --- |
|  |  | Assigned facility | | | | |
| Registered facility | Facility type | DH(%) | CHC(%) | PHC(%) | HWC(%) | Total (%) |
|  | DH | 22,582 (79.7) | 458 (1.6) | 1,367 (4.8) | 3,941 (13.9) | 28,348 (10.3) |
|  | CHC | 1,388 (2.8) | 36,582 (74.9) | 1,166 (2.3) | 9,700 (19.9) | 48,836 (17.9) |
|  | PHC | 660 (0.6) | 541 (0.5) | 76,602 (74.8) | 24,548 (23.9) | 102,351 (37.4) |
|  | HWC | 285 (0.3) | 596 (0.6) | 1,408 (1.5) | 91,531 (97.5) | 93,820 (34.3) |
|  | Total (%) | 24,915 (9.2) | 38,177 (14) | 80,543 (29.5) | 129,720 (47.5) | 273,355 (100) |
| DH, District Hospital; CHC, Community Health Center; PHC, Primary Health Center; HWC, Health and Wellness Center | | | | | | |
