# Supplementary material for: India Hypertension Control Initiative: decentralization of hypertension care to health wellness centres in Punjab and Maharashtra, India, 2018–2022
Source: BMC Health Serv Res. 2024 Aug 2;24:884. doi: 10.1186/s12913-024-11354-9 (PMC11297667; doi:10.1186/s12913-024-11354-9)
Supplement: Supplementary file 3 — Supplementary Material 3. [file 12913_2024_11354_MOESM3_ESM.docx]

| **Additional file 3: Treatment outcomes by facility type, Punjab and Maharashtra, India, 2019-22** | | | | | | | | | | | | |
| --- | --- | --- | --- | --- | --- | --- | --- | --- | --- | --- | --- | --- |
| **Blood pressure control %** | | | | | | | | | | | | |
| **Year** | **Under care (N)** | **2019** | **%** | **Under care (N)** | **2020** | **%** | **Under care (N)** | **2021** | **%** | **Under care (N)** | **2022** | **%** |
| **DH** | 5615 | 898 | 16 | 28124 | 8116 | 29 | 26021 | 11999 | 46 | 24915 | 11234 | 45 |
| **CHC** | 10031 | 2122 | 21 | 36718 | 13233 | 36 | 43311 | 20206 | 47 | 38177 | 17426 | 46 |
| **PHC** | 4379 | 982 | 22 | 60433 | 20252 | 34 | 75865 | 39302 | 52 | 80543 | 46573 | 58 |
| **HWC** | 322 | 2 | 1 | 20591 | 8305 | 40 | 45283 | 27384 | 60 | 129720 | 82362 | 63 |
| **Blood pressure uncontrol %** | | | | | | | | | | | | |
| **Year** | **Under care (N)** | **2019** | **%** | **Under care (N)** | **2020** | **%** | **Under care (N)** | **2021** | **%** | **Under care (N)** | **2022** | **%** |
| **DH** | 5615 | 1012 | 18 | 28124 | 6411 | 23 | 26021 | 5770 | 22 | 24915 | 6009 | 24 |
| **CHC** | 10031 | 1798 | 18 | 36718 | 8561 | 23 | 43311 | 10015 | 23 | 38177 | 7825 | 20 |
| **PHC** | 4379 | 803 | 18 | 60433 | 10124 | 17 | 75865 | 11781 | 16 | 80543 | 9730 | 12 |
| **HWC** | 322 | 4 | 1 | 20591 | 3649 | 18 | 45283 | 5022 | 11 | 129720 | 14321 | 11 |
| **Missed visit %** | | | | | | | | | | | | |
| **Year** | **Under care (N)** | **2019** | **%** | **Under care (N)** | **2020** | **%** | **Under care (N)** | **2021** | **%** | **Under care (N)** | **2022** | **%** |
| **DH** | 5615 | 3649 | 65 | 28124 | 13437 | 48 | 26021 | 6730 | 26 | 24915 | 7253 | 29 |
| **CHC** | 10031 | 6092 | 61 | 36718 | 14654 | 40 | 43311 | 12355 | 29 | 38177 | 12263 | 32 |
| **PHC** | 4379 | 2337 | 53 | 60433 | 29505 | 49 | 75865 | 22216 | 29 | 80543 | 21384 | 27 |
| **HWC** | 322 | 316 | 98 | 20591 | 8545 | 41 | 45283 | 11577 | 26 | 129720 | 29994 | 23 |

DH-District Hospital, CHC-Community Health Center, PHC- Primary Health Center, HWC- Health and Wellness Center
