# Supplementary material for: India Hypertension Control Initiative: decentralization of hypertension care to health wellness centres in Punjab and Maharashtra, India, 2018–2022
Source: BMC Health Serv Res. 2024 Aug 2;24:884. doi: 10.1186/s12913-024-11354-9 (PMC11297667; doi:10.1186/s12913-024-11354-9)
Supplement: Supplementary file 4 — Supplementary Material 4. [file 12913_2024_11354_MOESM4_ESM.docx]

**Additional Table 4: Association between age group and uncontrolled blood pressure, Punjab and Maharashtra, India, 2019-22**

|  |  | Uncontrolled | | Controlled | |  |  |  |  |  |  |
| --- | --- | --- | --- | --- | --- | --- | --- | --- | --- | --- | --- |
|  |  | n | % | n | % | RR | 95% CI | | aRR* | 95% CI | |
| Age (completed years) | <45 | 11,136 | 24.3 | 34,660 | 75.7 | ref. |  |  | ref. |  |  |
|  | 45-54 | 30,110 | 24.7 | 91,717 | 75.3 | 1.01 | 0.99 | 1.03 | 1.00 | 0.99 | 1.03 |
|  | 55-69 | 78,493 | 25.4 | 2,30,298 | 74.6 | 1.04 | 1.02 | 1.06 | 1.03 | 1.01 | 1.05 |
|  | >70 | 39,842 | 25.5 | 1,16,549 | 74.5 | 1.04 | 1.02 | 1.06 | 1.00 | 0.99 | 1.02 |

DH, District Hospital; CHC, Community Health Centre; PHC, Primary Health Centre; HWC, Health and Wellness Centre; SC, Sub-centre; CI, Confidence Interval; aRR, Adjusted Relative Risk. *adjusted for year of assessment, facility type, gender, baseline hypertension control status, diabetes, prior heart attack, prior stroke, prior CKD, taking hypertension drug at registration and state.
